# Antimicrobial usage and resistance in scottish dairy herds: a survey of farmers’ knowledge, behaviours and attitudes

**DOI:** 10.1186/s12917-023-03625-0

**Published:** 2023-05-19

**Authors:** Elena Borelli, Kathryn Ellis, Martin Tomlinson, Emily Hotchkiss

**Affiliations:** School of Biodiversity, One Health and Veterinary Medicine, Scottish Centre for Production Animal & Food Safety, University of Glasgow, 464 Bearsden Rd, Bearsden, Glasgow, G61 1QH UK

**Keywords:** Antimicrobial resistance, Dairy farmers, Scotland, Survey, Antimicrobial usage

## Abstract

Antimicrobial resistance (AMR) poses a major threat to global health. Understanding how antimicrobials are used on dairy farms and stakeholder beliefs relating to their use is essential to ensure responsible antimicrobial usage (AMU) to tackle the emergence of AMR. This study explored Scottish dairy farmers’ knowledge about the meaning of AMR and antimicrobial activity, behaviour and practices related to farm AMU and attitudes towards AMR mitigation. An online survey was designed based on the findings of two focus groups and was completed by 61 respondents (7.3% of the total population of Scottish dairy farmers). Knowledge of antimicrobials and AMR was variable, and almost half of the participants believed that antimicrobials could have anti-inflammatory or analgesic activity. Veterinarians’ opinions and advice about AMU were ranked significantly more important than other social referents or advisors. The majority of farmers (90%) reported having implemented practices to reduce reliance on antimicrobials (e.g., selective dry cow therapy, AMU treatment protocols) and having reduced farm AMU over recent years. Feeding waste milk to calves is still widespread, being reported by up to 30% of respondents. The main factors described to hinder responsible farm AMU were limited facilities (e.g., lack of isolation pens for sick animals) and knowledge of appropriate AMU recommendations, followed by time and financial constraints. Most farmers (89%) agreed that it is important to reduce AMU on dairy farms, but fewer (52%) acknowledged that AMU on UK dairy farms is currently too high, suggesting a mismatch between their intention to reduce antimicrobials and AMU behaviour. These results indicate that dairy farmers are aware of AMR, and their self-reported farm AMU has been reduced. However, some do not clearly comprehend the activity of antimicrobials and their correct usage. More work is needed to improve dairy farmers’ knowledge of appropriate AMU and intentions to combat AMR. Farmers would benefit from more regular AMU discussions and advice from herd veterinarians, as they were described as highly trusted information resources. Training on how to reduce AMU should involve all farm staff administering antimicrobials and should be tailored to farm-specific barriers, such as limited facilities and workforce shortages.

**Supplementary Information:**

The online version contains supplementary material available at 10.1186/s12917-023-03625-0.

## Introduction

Antimicrobial resistance (AMR) has been declared one of the top ten public health threats facing humanity [1]. Two major drivers for acquired AMR are excessive use and misuse of antimicrobials in humans and animals [2]. The emergence of resistant bacteria may lead to increased morbidity and mortality in cattle, resulting in compromised animal welfare and economic losses [3]. In addition, scientific evidence has indicated that antimicrobial usage (AMU) in animals may contribute to AMR in humans [4], and food-producing animals can be a reservoir of resistant pathogenic strains of some zoonotic bacteria such as *Staphylococcus aureus*, *Escherichia coli, Listeria monocytogenes*, and *Salmonella* spp. [5]. Based on the precautionary principle, it is necessary to address irresponsible AMU in food-producing animals to prevent potential adverse animal and public health consequences.

In dairy herds, antimicrobials are frequently used to treat infections in adult and young animals, with the control of udder infection representing the main reason for AMU [6]. In the UK, antimicrobials are a prescription-only medicine prescribed by a veterinary surgeon after a clinical assessment of the animal [7]. However, farmers often store and administer antimicrobials without the guidance of a veterinarian. Farm AMU is usually estimated from veterinary practices’ sales data or on-farm records [8]; however, monitoring AMU alone gives little evidence about farmers’ beliefs and intentions to reduce AMU. Understanding how antimicrobials are used in livestock is crucial for the development of antimicrobial stewardship programmes. Therefore, significant literature exists regarding what influences farmers’ behaviour and attitudes towards AMR, high-risk practices, and barriers and drivers for responsible AMU.

Along with the major international health organisations, many countries have developed regulations and directives on the prudent usage of antimicrobials in veterinary medicine. Recently, the UK government issued the most recent UK five-year national action plan, “Tackling antimicrobial resistance 2019–2024”, which focuses on reducing the need for antimicrobials through good husbandry, disease prevention, and biosecurity [9]. The Responsible Use of Medicines in Agriculture Alliance (RUMA) has formulated many guidelines for farmers and veterinarians regarding prudent AMU [10]. In addition, many dairy product purchasing companies are now requiring their supplier farms to demonstrate responsible AMU [11]. Yet, little is known about the uptake of the guidelines and the implementation of best practice recommendations on farms.

Previous studies in the UK report that farmers are generally aware of the emergence of resistant bacteria and recognise their responsibility to reduce AMU; however, there are other priorities than AMR in their everyday decision-making [12]. Dairy farmers often express concerns about the consequences of restricting AMU on productivity and animal welfare [12]. Also, they indicated several barriers to AMU reduction at the farm level, such as economic challenges, lack of skills and inadequate knowledge of the guidelines [12–14].

To the authors’ knowledge, no work to date has been carried out exploring stakeholder knowledge, behaviour, and attitudes relating to AMU and AMR in the Scottish dairy sector. Therefore, the objectives of this study were to investigate Scottish dairy farmers’ knowledge of antimicrobials and the meaning of AMR, the uptake of best practices to fight AMR, whether farm AMU has changed, and how they foresee it changing in future years. In addition, we aimed to assess dairy farmers’ attitudes towards AMR mitigation and the drivers, barriers, and facilitators to responsible farm AMU.

## Materials and methods

### Study population

A cross-sectional online survey was conducted in Scotland between the 26th of April and the 31st of August 2021. The survey URL was promoted in multiple ways (via the farming press, social media, veterinary practices, and milk buyers), and participation in the study was on a voluntary basis. The target population included all Scottish dairy farmers. Inclusion criteria were working on a Scottish dairy farm and being responsible for antimicrobial administration. Due to some specific dairy questions (e.g., milk production, somatic cell count), it would not have been possible for non-dairy farmers to complete the survey. According to the Scottish Dairy Cattle Association, there were 832 dairy farms in Scotland in 2021 [15]. Participants were provided with a Participant Information and Consent Form at the start of the survey. Although the survey was anonymous and did not collect personal information, farmers could disclose their e-mail addresses once the survey was submitted to participate in a prize draw to win one of four £25 Lidl vouchers, which was included as an incentive to complete the survey. The email address was solely used for the purpose of the prize draw. Findings from a focus group and workshop were used in the development of the survey. This research gained ethical approval from the local ethics committee (the College of Medical, Veterinary and Life Sciences, University of Glasgow).

### Focus group and workshop

As a result of the COVID-19 pandemic, the focus group and workshop were conducted remotely. The four authors attended and facilitated the discussion at both events. The focus group was held in August 2020 and included a convenience sample of dairy farmers personally known by the authors (n = 5). A PowerPoint presentation was used as a visual aid displaying some images (e.g., milk samples) and specific questions which are listed in Appendix I. The workshop was part of an online agricultural event (Agriscot 2020) held in November 2020, during which the authors presented some questions (Appendix I) via multiple polls to online participants (approximately n = 40). Only Scottish dairy farmers were encouraged to answer. The responses were displayed and used to elicit discussion, including via the chat function.

Some of the findings collected were used for the design of the survey. For instance, examples given by participants were used as options in the multiple-choice questions (e.g., best practices implemented on farms, barriers/drivers for AMU reduction).

### Survey design

The survey was devised using the Online Surveys tool for which the University of Glasgow holds an institutional license (JISC survey) and was structured in four main sections. It consisted of multiple-choice, matrix, ordinal, and open-ended questions to explore farmers’ knowledge, reported behaviours, and attitudes toward AMR. Antimicrobials discussed in the manuscript refer only to antibiotics.

Section One explored farmers’ awareness and understanding of AMR, the importance of the opinion of some social referents (e.g., veterinarian, milk buyer, consumers, other farmers) regarding their AMU, and their contact with veterinarians.

Section Two included questions investigating farmers’ AMU and implementation of practices to reduce reliance on antimicrobials. In addition, they were asked how their AMU has changed over the last few years and how they foresee it changing over the next five years. In this section, seven common dairy cattle disease scenarios (clinical mastitis, metritis, calf diarrhoea and pneumonia, lameness, drop in milk production) were described to assess the likelihood of farmers administering antimicrobials and following best practices (e.g., record keeping, waste milk disposal, duration of antimicrobial treatment, and biocontainment measures) (Table 1).

**Table 1:** Description of seven clinical disease scenarios typically encountered on dairy farms, included as part of a survey of Scottish dairy farmers

Scenario	Description
1	Milking cow: signs of mild mastitis (milk modified, udder inflamed, no fever, no systemic symptoms)
2	1-week-old calf: diarrhoea, no fever, slightly dehydrated, normal appetite
3	Cow: 10 days post-partum, smelly uterine discharge, temperature 39.5 °C
4	Six calves aged 1–2 months: cough, nasal discharge, fever (temperature > 39.5 °C)
5	Diarrhoea in 20% of young calves (1–3 weeks old) over the last month and few of them died
6	Milking cow: sudden lameness in one hind limb
7	Milking cow: sudden milk drop and fever (temperature = 39.9 °C)

In Section Three, participants were asked to rate attitudinal statements on a five-point Likert scale (from strongly disagree to strongly agree). Statements were created based on the authors’ experience and focus group/workshop findings, and explored farmers’ concerns and opinions about AMR, the necessity to reduce AMU in livestock and its impact on public health, and the importance of some best practices on farms (e.g., record keeping, drug protocols, hospital pen).

Section Four gathered demographic and production information of the participants and the farm.

A pilot study was performed using a group of dairy farmers personally known by the authors (n = 5) to test the survey duration and suitability of the questions to the target population. The final survey included 54 main questions, with 33 of these being multiple choice, 13 Likert scale, five matrix, two open-ended, and one ordinal. In some cases, a comment field was provided to enter free text. Some questions were followed by sub-questions depending on the answer selected (skip pattern). With the exclusion of the name of the milk buyer and the personal e-mail address, it was required to answer all questions to submit the survey. The survey is available in Appendix II.

### Statistical analysis

Data were downloaded from the Online Surveys tool in an electronic Excel dataset format and were cleaned to remove potential missing or error data. Data analyses were performed using R Core Team (2020).

Demographic and herd production data of respondents were assessed to investigate whether the sample was representative of the target population with respect to herd size, somatic cell count (SCC), and milk yield (Two-sample Kolmogorov-Smirnov test). Reference herd size data were obtained from the Scottish Dairy Cattle Association, while SCC and milk yield information was provided by the Cattle Information Service (CIS) in Scotland.

### Descriptive statistics

Descriptive statistics of responses (frequencies and proportions) were analysed for each question. Continuous variables (herd average SCC, milk yield, and the number of milking cows) were tested for normality with the Shapiro test and described with mean or median, minimum, and maximum. Responses to the open-ended knowledge question (“What does AMR mean to you in your own words?”) were categorised into common themes based on the respondent’s interpretation.

### Visual representation and statistical test of likert and matrix responses

Responses to matrix questions and attitudinal five-point Likert scale were represented with stacked bar charts. Differences between the ranking of the factors were compared with the Kruskal Wallis test, followed by pairwise comparisons with the Wilcoxon test and Benjamini and Hochberg correction. Attitude responses were also tested for internal reliability with the aid of Cronbach’s alpha coefficient.

### Univariable associations

Univariable associations between categorical variables of interest were tested with the Pearson chi-squared test or Fisher exact test, and significant associations were reported in the manuscript. With this aim, the four categories of RUMA guideline familiarity (Q.5 and Q.5.a) were combined into three levels: low (answers “never heard” and “not familiar at all”), medium (answer “somewhat familiar”), and high familiarity (answer “very familiar”). The level of agreement with the statement “I am worried about AMR on UK dairy farms” (Q.44) was categorised into three groups: disagree (answers “strongly disagree” and “disagree”), neutral (answer “neither disagree nor agree”) and agree (answers “agree” and “strongly agree”). The frequency of veterinarian discussion about AMR (Q.9 and Q.9.a) was categorised into three levels: never (answer “never discussed”), once a year (answer “annually”) and twice a year or more (answers “every six months”, “monthly” and “at every visit”). The level of significance was set at P < 0.05.

## Results

In total, 61 respondents completed the survey, accounting for 7.3% of the 2021 population of Scottish dairy farmers. All versions were answered completely, and none were excluded from the analysis.

### Demographic data

Demographic data are summarised in Table 2. Most respondents were male (90%). The most frequent highest-level of education reported was agricultural college (52%), followed by university (33%) and high school (15%). Almost half of the farmers were between 36 and 50 years old and had between 21 and 40 years of experience in farming. Farm owners represented 72% of respondents, whilst the rest were employees (dairy managers). Most farms (93%) were conventional (not organic). Over half (52%) were breeding only dairy cows, while the rest included beef and/or sheep farming. Regarding infectious disease status, 10% of the herds did not have any disease-free accreditation, almost half (48%) reported only Bovine Viral Diarrhoea (BVD) free accreditation, while the remainder had other disease-free accreditation in addition to BVD. Median herd size (milking and dry cows) was 192 adult cows (range 46-1790); herd mean 305d milk production per cow was 8,999 Kg (range 5000–16,000 Kg); herd mean SCC was 135,000 cells/ml (range 56,000-215,000 cells/ml). Sample herd size, milk production and average SCC were representative of the general population of Scottish dairy farms (P > 0.05, Two-sample Kolmogorov-Smirnov test).

**Table 2:** – Results of a survey of Scottish dairy farmers - farmer demographics and farm characteristics

Q	Indicators	Categories	N	%
45	Age	18–35 36–50 > 51	13 28 20	21 (13/61) 46 (28/61) 33 (20/61)
46	Sex	Male Female	55 6	90 (55/61) 10 (6/61)
47	Years in farming	< 5 6–20 21–40 > 41	0 20 26 15	0 (0/61) 33 (20/61) 43 (26/61) 25 (15/61)
48	Education level	High school Agricultural college University	9 32 20	15 (9/61) 52 (32/61) 33 (20/61)
49	Farm system	Conventional Organic	57 4	93 (57/61) 7 (4/61)
51	Disease free accreditation	None Only BVD^a^ BVD^a^ plus other (IBR^b^/Leptospirosis)	6 29 26	10 (6/61) 48 (29/61) 42 (26/61)
52	Cattle bought last year	No Yes	37 24	61 (37/61) 39 (24/61)
53	Farm type	Only dairy Dairy and beef Dairy and sheep Dairy, sheep, and beef	32 9 10 10	52 (32/61) 15 (9/61) 16 (10/61) 16 (10/61)
54	Role	Owner Dairy manager	44 17	72 (44/61) 28 (17/61)

^a^ Bovine viral diarrhoea

^b^ Infectious bovine rhinotracheitis

### Section one (knowledge: farmer awareness and understanding of AMR)

Farmer understanding of AMR (open-ended question) varied: around a third gave a correct interpretation referable to “bacteria develop resistance and do not respond to some antimicrobials”, while the remaining indicated AMR as a “loss of efficacy of antimicrobials” (e.g., *“antibiotics are not working anymore”*) or “resistance to the drug developed by animals” (e.g., *“animals become immune to antibiotics*”) (Table 3). All farmers answered that antimicrobials are effective against bacteria, although 31% believed they could also be effective against viruses and 25% against parasites. Almost half of the respondents thought that antimicrobials have an anti-inflammatory and/or analgesic effect. The majority (92%) had discussed AMR with their veterinarians. This happened generally once a year for half of them, while the remaining reported a higher frequency (twice a year or more). Respondents showed good awareness of RUMA, with 90% having heard about the guidelines before. Of them, 71% and 22% reported being moderately familiar and very familiar with the recommendations. It was found that perceived knowledge of RUMA recommendations was significantly higher in owners than employees (P < 0.05, Pearson’s chi-squared test).

**Table 3:** Results of a survey of Scottish dairy farmers - farmer awareness and understanding of antimicrobial resistance (AMR).

Q	Indicators	Categories	N	%
2	AMR definition (open question)	‘Resistance to antibiotic developed by animals’ ‘Loss of efficacy of antibiotics’ ‘Resistance to antibiotic developed by pathogens’	17 23 21	28 (17/61) 38 (23/61) 34 (21/61)
3	Antibiotics are effective against	Bacteria Virus Parasite	61 19 15	100 (61/61) 31 (19/61) 25 (15/61)
4	Activity of antibiotic	Only anti-bacterial Anti-bacterial and anti-inflammatory and/or analgesic	32 29	52 (32/61) 48 (29/61)
5	Heard about RUMA^a^ before	Yes No	55 6	90 (55/61) 10 (6/61)
5.a	Familiarity with RUMA^a^	Not familiar at all Somewhat familiar Very familiar	4 39 12	7 (4/55) 71 (39/55) 22 (12/55)
6	Frequency of routine visit in the last month	Never Once More than once Once every week/more	9 21 15 16	15 (9/61) 34 (21/61) 25 (15/61) 16 (16/61)
7	Required an emergency visit in the last month	Never Once More than once Once every week/more	26 24 6 5	43 (26/61) 39 (24/61) 10 (6/61) 8 (5/61)
9	Antibiotic resistance previously discussed with veterinarian	Yes No	56 5	92 (56/61) 8 (5/61)
9.a	Frequency of antibiotic resistance discussion with veterinarian	Annually Every six months Monthly At every visit	28 21 7 0	50 (28/56) 37 (21/56) 13 (7/56) 0 (0/56)
8	Veterinarian consultation before antibiotic usage	Never Sometimes Most of the time Always	1 43 11 6	2 (1/61) 70 (43/61) 18 (11/61) 10 (6/61)
16	Main reason for veterinarian consultation before antibiotic usage	Economic value of animal Previous treatment unsuccessful Animal welfare Several animals involved	27 17 15 1	45 (27/60) 28 (17/60) 25 (15/60) 2 (1/60)
17	Main reason for not consulting the veterinarian before antibiotic usage	I have enough experience Cost Delay in treating animal Additional work	26 17 12 0	47 (26/55) 31 (17/55) 22 (12/55) 0 (0/55)

^a^ Responsible Use of Medicines in Agriculture Alliance

**Fig. 1:**
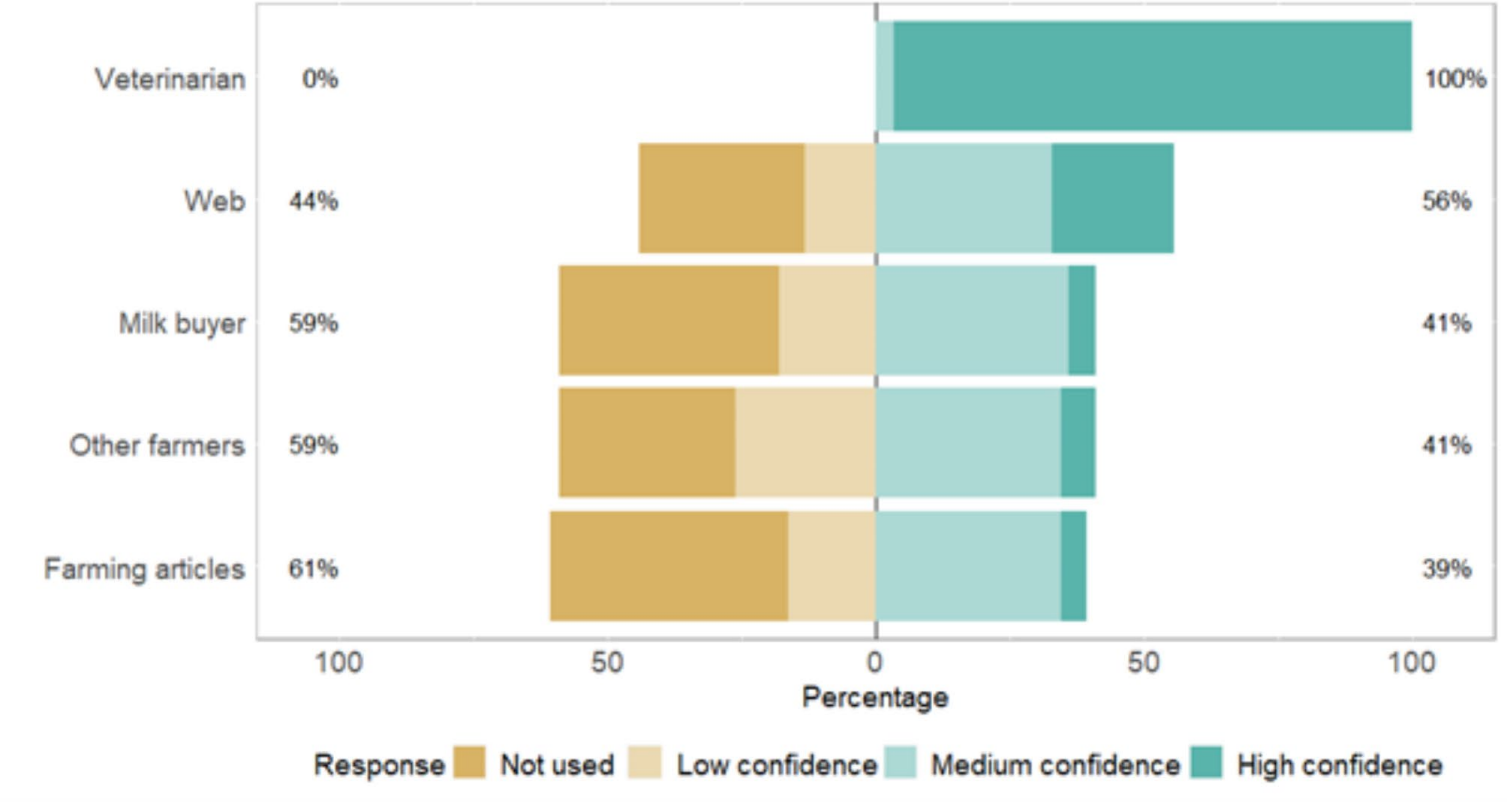
The confidence in different sources of information used by Scottish dairy farmers regarding responsible antimicrobial usage. The proportions of farmers ranking each source of information as not used/used with low confidence (scores 1 and 2) or used with medium confidence/used with high confidence (scores 3 and 4) are indicated

Farmers indicated which sources they consult for AMU guidance and to what extent they trust the information (Fig. 1). Veterinarian information was ranked significantly more reliable than other sources (P < 0.001, Kruskal-Wallis), followed by milk buyer guidance. Web, farming articles, and other farmers’ information were considered less trustworthy sources of information.

Around two-thirds of farmers reported occasionally consulting their veterinarian before using antimicrobials, whilst the remainder asks for advice most of the time (18%) or always (10%). Only one farmer admitted to never seeking a veterinarian’s opinion before AMU. High economic value of the animal represented the main reason for a veterinary consultation (27/60, 45%), followed by failure of previous treatment (17/60, 28%) and concerns over animal welfare (15/60, 25%). One farmer consulted veterinarians primarily when multiple animals are affected. Sufficient perceived personal experience was the main reason for not calling the veterinarian (26/55, 47%), followed by cost (17/55, 31%) and delay in treatment (12/55, 22%).

**Fig. 2:**
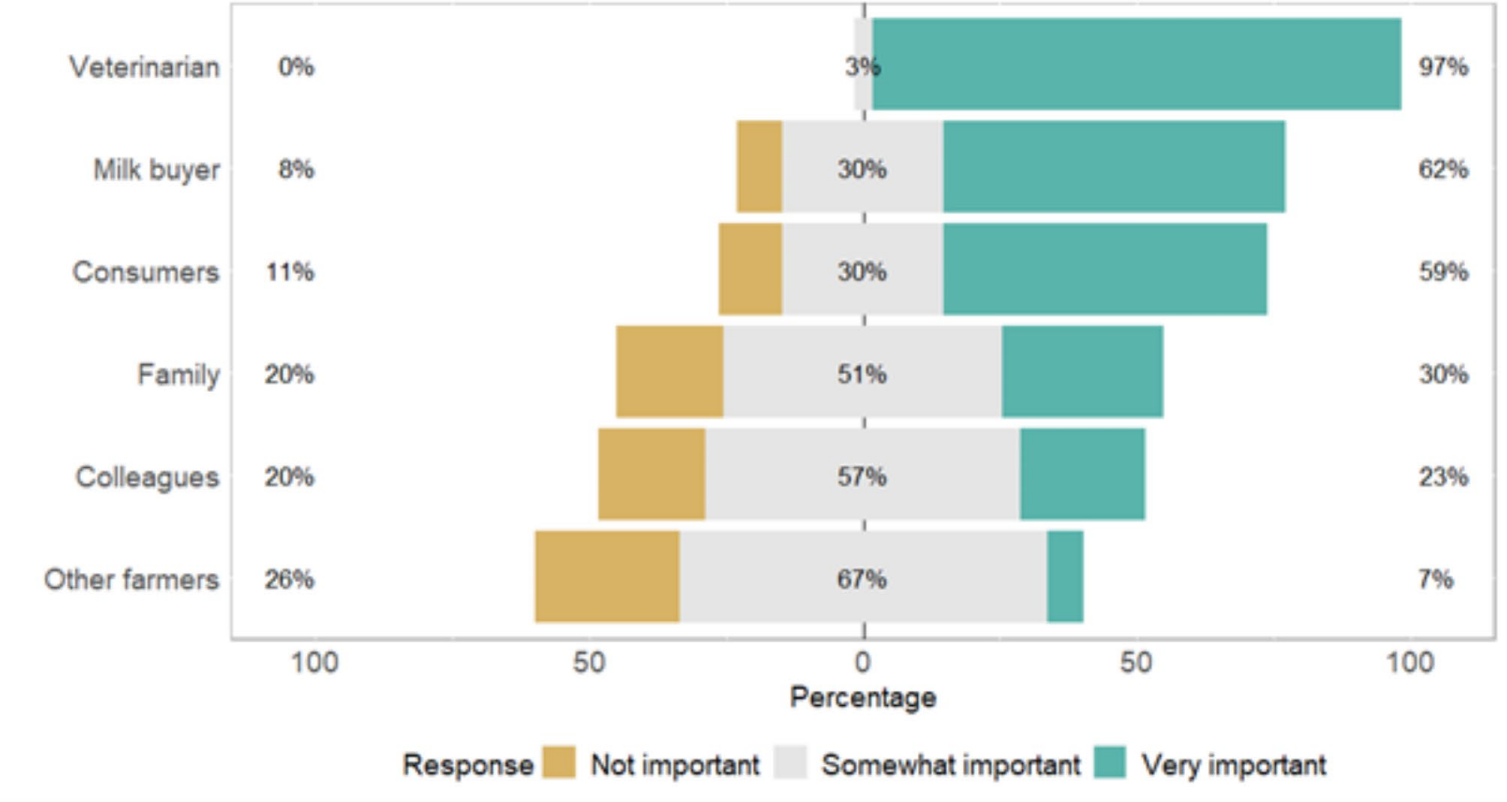
The importance of the opinion of some social referents for Scottish dairy farmers regarding their antimicrobial usage. The proportions of farmers ranking the opinion of each social referent as not important (score 1), somewhat important (score 2), and very important (score 3) are indicated

Farmers were questioned on the importance of the opinion of some social groups regarding their AMU (Fig. 2). Their veterinarian’s opinion was significantly more important than others’ opinions (P < 0.001, Kruskal-Wallis). It was followed by milk buyers’ and consumers’ opinions, which were ranked significantly higher than family, colleagues, and other farmers’ opinions (P < 0.001, Kruskal-Wallis).

### Section two (reported behaviours: antimicrobial usage on farm)

**Fig. 3:**
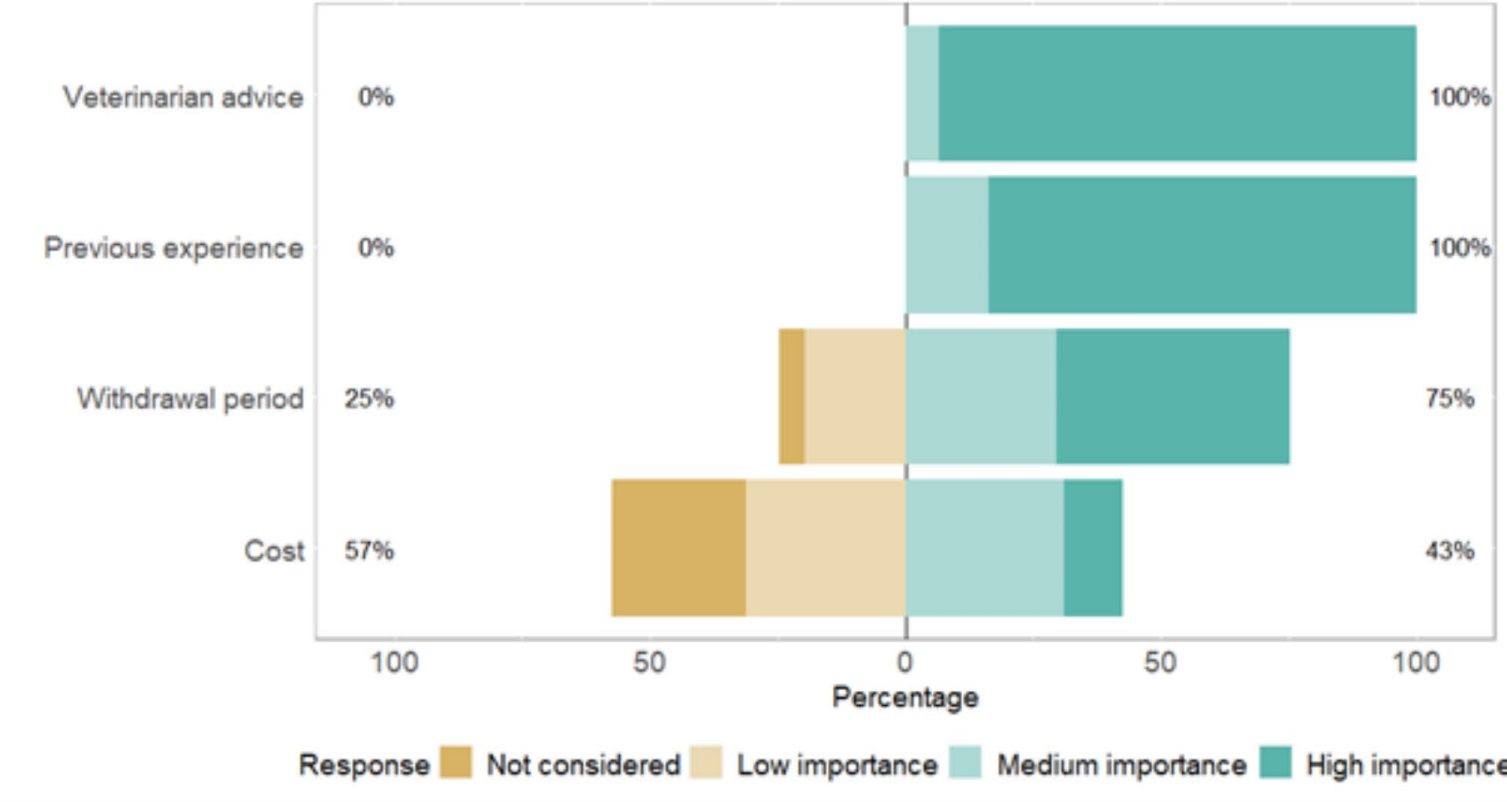
The importance of factors considered for antimicrobial choice by Scottish dairy farmers. The proportions of farmers ranking each factor as not considered/considered with low importance (scores 1 and 2) or considered with medium importance/considered with high importance (scores 3 and 4) are indicated

Farmers indicated to which extent some factors influence their antimicrobial choice (Fig. 3). Veterinary advice and previous usage experience were ranked as more important than other factors in medicine choice (P < 0.001, Kruskal-Wallis). The withdrawal period was more important than the cost (P < 0.001, Kruskal-Wallis).

Table 4 summarises the results of farmers’ AMU behaviour. Farmers ranked a list of common diseases from the most frequent (1st) to the least frequent (6th) reason for antimicrobial administration. Overall, udder health was ranked first by 80% of the farmers, with 59% indicating “mastitis” and 21% indicating “dry cow therapy” as the main drivers for AMU. Calf pneumonia was another important reason for AMU, ranked first by 15% of the farmers. Post-partum disease, calf diarrhoea, and lameness were less frequently reported as the primary cause of AMU. Penicillin was the antimicrobial most commonly used by 85% of farmers.

**Table 4:** Results of a survey of Scottish dairy farmers - reported antimicrobial usage (AMU) and practices on farm

Q	Indicators	Categories	N	%
12	Most frequently used antibiotic	Penicillin Oxytetracycline Ceftiofur Tylosin	52 6 2 1	85 (52/61) 10 (6/61) 3 (2/61) 2 (1/61)
13	Main condition treated with antibiotic	Mastitis Dry cow therapy Calf pneumonia Post-partum disease Calf diarrhoea Lameness	36 13 9 4 2 1	59 (36/61) 21 (13/61) 15 (9/61) 6 (4/61) 3 (2/61) 2 (1/61)
14	Practices on farm to reduce antibiotic usage	Yes No	55 6	90 (55/61) 10 (6/61)
14	Practices to reduce antibiotic usage mentioned in the open question	SDCT^a^ Hygiene/comfort Vaccination Probiotics Footbath Milk culture	38 12 7 4 2 2	69 (38/55) 22 (12/55) 13 (7/55) 7 (4/55) 4 (2/55) 4 (2/55)
15	Antibiotic treatment protocols on farm	Yes No, planning to have in future No and no intention to have	41 16 4	67 (41/61) 26 (16/61) 7 (4/61)
19	Culture and sensitivity of samples	Regularly Occasionally Never	14 37 10	23 (15/61) 61 (37/61) 16 (10/61)
19.a	Reason for not using culture and sensitivity regularly	Delay of the results Uncertainty about the benefit Inconclusive results Cost	34 14 6 3	72 (34/47) 30 (14/47) 13 (6/47) 6 (3/47)
20	SDCT^a^ implemented on farm	Yes No, considering in the future No and no intention to have	53 4 4	86 (53/61) 7 (4/61) 7 (4/61)
20	Proportion of cows receiving antibiotic at dry off (open question)	0% 1–10% 11 − 10% 21–30% 40–50% 100%	8 11 11 12 11 8	13 (8/61) 18 (11/61) 18 (11/61) 20 (12/61) 18 (11/61) 13 (8/61)
21	Antibiotic usage change last years	Less Same More	55 6 0	90 (55/61) 10 (6/61) 0 (0/61)
21.a	Reducing antibiotic usage was difficult	Yes No	27 28	49 (27/55) 51 (28/55)
21.b	Reducing antibiotic usage would be difficult	Yes No	3 3	50 (3/6) 50 (3/6)
21.a.b	Main barriers to reduce antibiotic usage (open question)	Limited facilities Lack of knowledge Increased labour Limited finances Welfare/productivity concern Lack of staff compliance	11 9 7 7 6 3	37 (11/30) 30 (9/30) 23 (7/30) 23 (7/30) 20 (6/30) 10 (3/30)
22	Antibiotic usage change next 5 years	Less Same More	53 8 0	87 (53/61) 13 (8/61) 0 (0/61)

^a^ Selective dry cow therapy

**Fig. 4:**
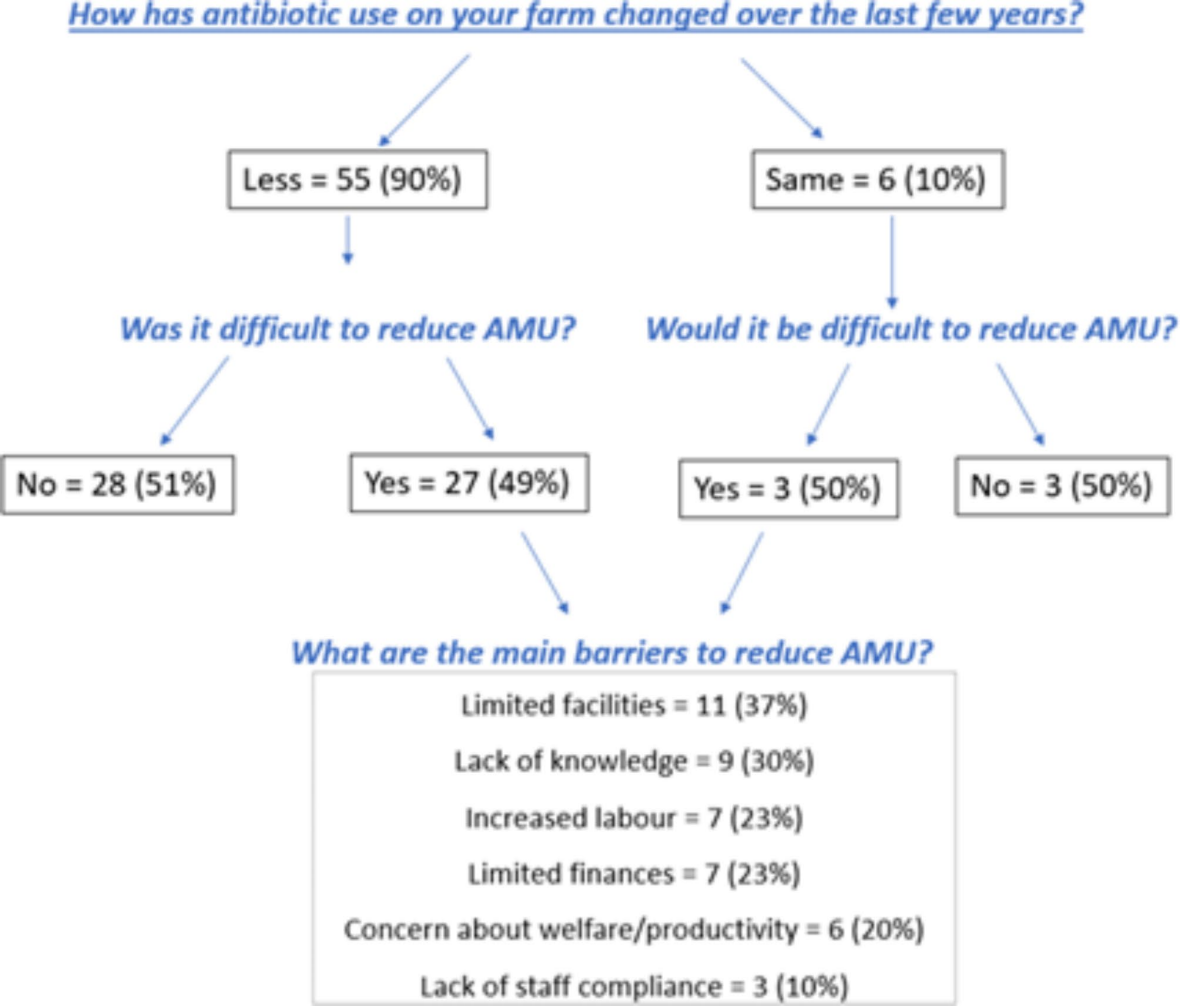
Antimicrobial usage (AMU) change in the last few years and main barriers associated with reduced AMU for Scottish dairy farmers

Most farmers (90%) reported having implemented practices to reduce farm AMU, and the same proportion reported having decreased their AMU in recent years. Half of the farmers thought limiting their AMU was difficult, with limited facilities and lack of knowledge being the main barriers (Fig. 4). Regarding future intention, 87% of farmers planned to decrease farm AMU in the next five years.

When asked to give examples of the practices implemented via an open question, selective dry cow therapy (SDCT) was the most cited (38/55, 69%), followed by improved hygiene and comfort and vaccination. Around 67% reported having written antimicrobial treatment protocols, and 26% planned to introduce them in the future. Having treatment protocols was associated with a higher frequency of veterinarian discussion about AMR (P < 0.05, Pearson’s chi-squared test). Some farmers reported regularly sampling diseased animals (e.g., faeces or milk) for microbiological culture (23%), with the majority doing it only occasionally (61%). When questioned on reasons for not using culture, 34/47 (72%) indicated results being too slow, 14/47 (30%) doubted the benefits, and 6/47 (13%) claimed frequent inconclusive results. In contrast, only a few answers were related to a cost issue (3/47, 6%). Most farmers (86%) had implemented SDCT, among which eight did not use antimicrobials in any cows at dry-off. Around 7% were planning to introduce SDCT in the future, while the remainder reported no intention to implement it.

Two questions explored the main motivators and concerns associated with decreasing AMU. Minimising antimicrobial residues and meeting milk buyer standards had a major influence on reducing AMU in most of the respondents (75%). Only 36% indicated minimising cost as an important motivator to reduce AMU. Adverse effects on animal health and welfare were the main concerns associated with decreasing AMU. In contrast, potential economic consequences (e.g. decreased profitability, milk production, rising of other costs) were less worrying for respondents.

**Fig. 5:**
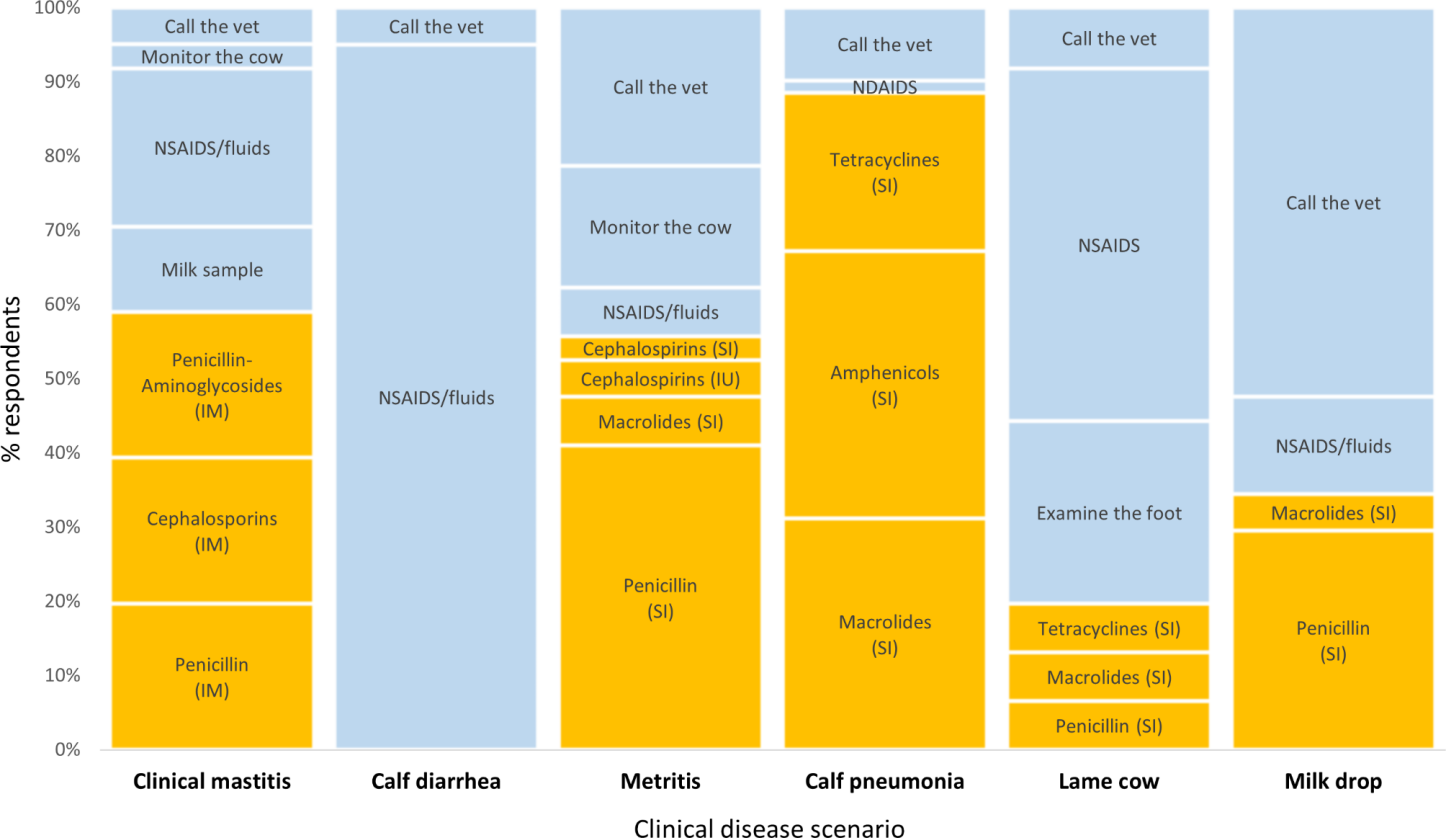
Proportion of Scottish dairy farmers who selected an antimicrobial treatment (orange) and proportion of Scottish dairy farmers who selected an alternative to antimicrobial treatment (blue) in the clinical disease scenarios

In the disease scenarios, antimicrobials were most frequently chosen as the first treatment option in cases of calf pneumonia (89%), followed by clinical mastitis (59%) and metritis (56%). Fewer farmers used antimicrobials for cow lameness (20%) and milk drop (e.g., a drop in milk production) (34%), and none for calf diarrhoea. Only approximately 10% of the farmers collected a milk sample before administering antimicrobials for mastitis. Some farmers opted for NSAIDS/fluids as the first treatment in all scenarios, with this proportion particularly high for calf diarrhoea (93%) and cow lameness (48%). The veterinarian consultation was selected by more than half of the participants for the scenario of milk drop and fever (Fig. 5).

Some best practices were tested in the clinical disease scenarios (Table 1). In the case of clinical mastitis and calf pneumonia, all respondents registered the treatment in the medicine book. While a computer was always used for recording clinical mastitis, it was used by only 13% of the farmers for calf pneumonia. In the case of clinical mastitis and metritis, around two-thirds of farmers did not use the milk of the treated cow to feed calves. Whilst in calf pneumonia approximately 30% of the farmers followed the treatment duration suggested in the farm protocols, this option was not selected for the lame cow scenario, indicating a potential absence of protocols for this clinical condition. In the case of the calf diarrhoea scenarios (scenarios 2 and 5), more than half (69%) isolated the sick animals; however, only 3% and 10% respectively fed them last. Approximately 84% chose to collect a faecal sample for culture. No farmers decided to use a prophylactic treatment in other calves.

### Section three (farmer attitudes: towards AMU and concern about AMR)

**Fig. 6:**
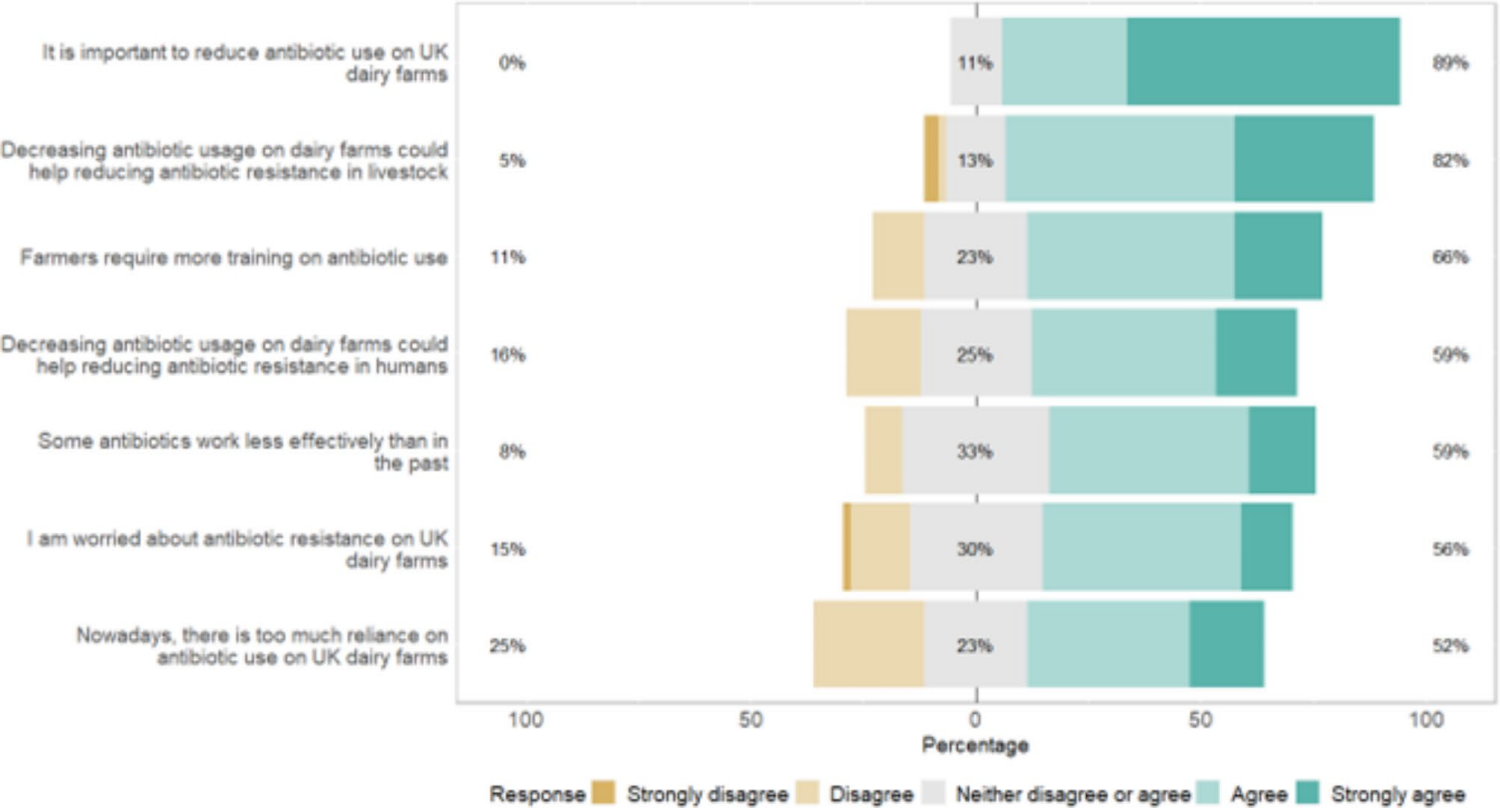
The level of agreement of Scottish dairy farmers with some statements regarding antimicrobial resistance in dairy farms. The proportions of farmers ranking the level of agreement with each statement as strongly disagree and disagree (scores 1 and 2), neither disagree nor agree (score 3), and agree and strongly agree (scores 4 and 5) are indicated (UK, United Kingdom)

Figure 6; Table 5 show the level of agreement with some attitude statements about AMR. Most farmers (89%) believed that reducing AMU on UK dairy farms is important. In comparison, there was significantly less agreement with there being too much reliance on antimicrobials, or with the statement “I am concerned about AMR in the UK dairy farms” (P < 0.001, Kruskal-Wallis). Most participants (82%) agreed that AMU on farms might contribute to the emergence of AMR in livestock. In comparison, there was significantly less agreement on the association with human AMR (P < 0.001, Kruskal-Wallis). These five attitude statements are related to the perception of AMR as a threat (Cronbach’s α factor = 0.80). Regarding farmers’ perceived ability, about 66% expressed the need for more training to reduce their AMU.

**Table 5:** Results of a survey of Scottish dairy farmers - attitudes towards antimicrobial resistance (AMR) and best practices implementation

Q	Indicators	Categories	N	%
32	It is important to reduce antibiotic usage on UK^a^ dairy farms	Strongly agree Agree Neither agree nor disagree Disagree Strongly disagree	37 17 7 0 0	61 (37/61) 28 (17/61) 11 (7/61) 0 (0/61) 0 (0/61)
33	Nowadays, there is too much reliance on antibiotic usage on dairy farms in the UK^a^	Strongly agree Agree Neither agree nor disagree Disagree Strongly disagree	10 22 14 15 0	16 (10/61) 36 (22/61) 23 (14/61) 25 (15/61) 0 (0/61)
34	Decreasing antibiotic usage in dairy farms could help reducing antibiotic resistance in livestock	Strongly agree Agree Neither agree nor disagree Disagree Strongly disagree	19 31 8 1 2	31 (19/61) 51 (31/61) 13 (8/61) 2 (1/61) 3 (2/61)
35	Decreasing antibiotic usage in dairy farms could help reducing antibiotic resistance in humans	Strongly agree Agree Neither agree nor disagree Disagree Strongly disagree	11 25 15 10 0	18 (11/61) 41 (25/61) 25 (15/61) 16 (10/61) 0 (0/61)
36	Some antibiotics work less effectively than in the past	Strongly agree Agree Neither agree nor disagree Disagree Strongly disagree	9 27 20 5 0	15 (9/61) 44 (27/61) 33 (20/61) 8 (5/61) 0 (0/61)
37	Farmers require more training on antibiotic usage	Strongly agree Agree Neither agree nor disagree Disagree Strongly disagree	12 28 14 7 0	20 (12/61) 46 (28/61) 23 (14/61) 11 (7/61) 0 (0/61)
38	Farm biosecurity and vaccination can reduce antibiotic usage	Strongly agree Agree Neither agree nor disagree Disagree Strongly disagree	29 27 5 0 0	48 (29/61) 44 (27/61) 8 (5/61) 0 (0/61) 0 (0/61)
39	It is important to have protocols for antibiotic usage on farm	Strongly agree Agree Neither agree nor disagree Disagree Strongly disagree	28 29 4 0 0	46 (28/61) 60 (29/61) 6 (4/61) 0 (0/61) 0 (0/61)
40	It is important to keep treatment records on farm and review antibiotic usage regularly	Strongly agree Agree Neither agree nor disagree Disagree Strongly disagree	41 20 0 0 0	67 (41/61) 33 (20/61) 0 (0/61) 0 (0/61) 0 (0/61)
41	It is important to always respect the prescribed duration course of antibiotic	Strongly agree Agree Neither agree nor disagree Disagree Strongly disagree	39 18 4 0 0	64 (39/61) 30 (18/61) 6 (4/61) 0 (0/61) 0 (0/61)
42	It is important to have hospital pens to isolate sick animals and avoid the spread of the diseases	Strongly agree Agree Neither agree nor disagree Disagree Strongly disagree	24 31 5 1 0	39 (24/61) 51 (31/61) 8 (5/61) 2 (1/61) 0 (0/61)
43	It is important to always respect the withdrawal period of treated animals before slaughter or including the milk in the bulk milk tank	Strongly agree Agree Neither agree nor disagree Disagree Strongly disagree	54 6 1 0 0	88 (54/61) 10 (6/61) 2 (1/61) 0 (0/61) 0 (0/61)
44	I am worried about antibiotic resistance on UK^a^ dairy farms	Strongly agree Agree Neither agree nor disagree Disagree Strongly disagree	7 27 18 8 1	11 (7/61) 44 (27/61) 30 (18/61) 13 (8/61) 2 (1/61)

^a^ United Kingdom

Farmers who described a correct definition of AMR expressed more concerns about AMR on UK dairy farms (p < 0.002, Pearson’s chi-squared test).

Regarding attitudes towards best practices, the majority of farmers (> 90%) agreed on the importance of all best practices presented: keeping records of AMU, respecting the withdrawal period and the prescribed duration of AM treatment, having treatment protocols, implementing farm biosecurity and vaccination to reduce AMU, and having an isolation pen for sick animals. These statements are related to the perception of the importance of best practices for responsible AMU (Cronbach’s α factor = 0.75).

## Discussion

Understanding how dairy farmers use antimicrobials, whether they are aware and concerned about AMR, and whether they are willing to change their practices is important for policymakers and farm advisors to develop effective strategies aimed at reducing AMU.

This study indicated that participants’ knowledge about antimicrobials and AMR was variable. Proper AMU and the significance of AMR might still be unknown for some dairy producers. Similar to the UK general population [16], approximately one-third of the farmers believed that antibiotics could be used to treat virus infections. Only 34% reported an accurate definition of AMR, a smaller proportion than previously indicated by an English dairy farmers’ survey where 55% provided a correct description of AMR [14]. The difference may be due to a stricter interpretation in our study, as only the definition of AMR as “bacteria developing resistance to antimicrobials” was considered correct. Despite the description given by some respondents as a “loss of efficacy of antibiotics” cannot be regarded as false, this represents a consequence of AMR and is not a correct definition.

Our study found that reported awareness of RUMA guidelines was higher than previously found by Jones et al. in a 2013 survey of English and Welsh producers [13]. A potential explanation may be a trend for farmers to be more exposed to AMU restrictions and regulations in recent years, although this may not translate into knowledge. Farm owners’ familiarity with the guidelines was greater than dairy herd managers. Farm employees are often the main ones responsible for administering antimicrobials, particularly in large-size herds. Our results suggest that in addition to the need for campaigns regarding responsible AMU in the Scottish dairy sector, knowledge and awareness should be disseminated consistently among all stakeholders handling antimicrobials. For instance, dairy managers should be involved in the farm AMU monitoring and the decision-making process of antimicrobial treatment protocols with the herd’s veterinarian. Also, farm employees involved with AMU should be encouraged to participate in responsible AMU training, such as the one certified by the Red Tractor [17].

Farmers’ implementation of advice depends on who delivers the recommendation [18]. Similar to previous studies [12, 13, 19–21], this survey shows that herd veterinarians are regarded as the most reliable source of information and the most influencing social referent. However, we found that discussions about AMR with the veterinarian were infrequent. For half of the respondents, it happens once a year, likely at the AMU review required by the Red Tractor Assurance, a UK food and farm standard voluntary scheme guaranteeing food safety, traceability, and animal welfare across livestock species [17]. The lack of communication around AMR may be due to the infrequent contact with veterinarians, as suggested by our results and other studies [13, 22]. Also, it could be linked to veterinarians’ perceptions that dairy producers are reluctant to change their practices [12, 23], and to veterinarians’ time limitations [24]. Since veterinarian recommendations have the most decisive influence on farmers’ behaviour, practitioners must be aware of their role in tackling AMR and should dedicate part of their work to this purpose. According to research conducted in the UK, despite antimicrobial stewardship principles being included in most undergraduate veterinary schools, there is still room for improvement in students’ education in this area [25]. Enhancing future veterinarians’ education is important to improve their prescribing behaviour and provide them with the skills to engage farmers in the fight against AMR. Also, continuing professional development (CPD) courses on this topic, including aspects of social science, may help practitioners to facilitate changes on farms.

Our results suggest that farmers tend to rely on personal experience when choosing antimicrobials and veterinarians are consulted only occasionally, although they are considered trusted advisors. The widespread implementation of written AMU protocols may partially explain this finding. Similarly, in previous studies, farmers reported relying significantly on pre-existing experiences and some even more than on veterinarians’ advice [26, 27]. Repeated positive outcomes of some antimicrobials may lead farmers to have preferred treatments that they consider more effective. Ritualising drug choice is likely to act as a barrier to farmers’ responsible AMU, as behavioural interventions are more difficult when there is an over-reliance on previous experiences [28]. Farmers are reluctant to implement new recommendations if they do not believe they are practical and feasible [29] it is essential to educate and promote the effectiveness of alternative and responsible practices to change deeply embedded behaviours. Strategies, such as showing results in other dairy farms, may help farmers to change their perceptions [18].

Previous research on dairy farmers’ AMU behaviour highlighted the importance of external approval and social norms conformity [13, 22, 30, 31]. Unlike other surveys [20, 22], farmers in our study did not express the need to be considered a “good farmer” by other producers and did not feel pressured by peers’ opinions (e.g., colleagues, family, and other farmers). Gerber and colleagues reported similar results [32], with most participants not motivated by other farmers to reduce their AMU. On the other hand, we found that farmers valued the opinion of some social referents in the dairy industry (e.g., milk buyers and consumers), and minimising residues and meeting milk buyer standards were the main drivers for lower AMU. Although some farmers may feel constrained by public demand for antibiotic-free products and the negative perception of the industry [20, 22], our results suggest a positive impact of consumer pressure in shaping and improving AMU in livestock. Since consistent findings were reported in other UK studies [12, 13], farmers’ attitudes towards consumers likely depend on the agricultural framework of the country.

Farmers indicated mastitis as the main reason for AMU and penicillin as the most used antimicrobials, also reported by previous studies in the UK and Scotland [14, 33]. Instead, in the scenarios section, we found that antimicrobials were most frequently chosen as a treatment option in calf pneumonia, followed by clinical mastitis. None of the respondents used antimicrobials for the diarrhoea calf scenario as a first-line treatment, suggesting a much lower AMU for this condition than in UK beef calves [21]. The different finding may be due to more extensive production in the beef sector, with less veterinarian contact and antimicrobial treatment protocols.

This survey showed that most farmers had already taken steps to reduce AMU: around 90% had practices implemented on farms, with SDCT being the most cited. The presence of antimicrobial treatment protocols was widespread among participants, and generally, these were more popular than in other studies [34, 35]. This may be explained by the larger size of the Scottish herds compared to other EU countries and by the compulsory requirement set by some UK milk buyers [15]. Culture and sensitivity of biological samples from diseased animals (e.g., faeces and milk) was reported to be used only occasionally and to a lesser extent than in other countries [35]. Interestingly, lab costs were a limiting factor for only a few farmers, whereas the main reason for not using this practice was the delay in the results. The clinical disease scenarios suggested that producers were more likely to collect a biological sample for culture in case of a disease outbreak (e.g., calf diarrhoea) rather than for diagnostic testing in an individual animal infection (e.g., mastitis). Clinical mastitis is usually treated symptomatically without knowing the aetiology, making administering antimicrobials often unnecessary [36]. Research reports that *E. coli* udder infection commonly cures spontaneously, 19–46% of clinical cases of mastitis are microbiologically negative, and some pathogens (e.g., yeasts, *Serratia* spp. etc.) do not respond to antimicrobial treatments [6]. Therefore, many cases of non-severe clinical mastitis would not benefit from antimicrobial therapy [37]. Farmers need to be trained to identify more precisely which animals require antimicrobial treatment and to ensure evidence-based AMU. Newer point-of-care diagnostics may overcome some of the limitations of classic laboratory tests and offer more rapid results [38].

Our study suggests that feeding waste milk to calves is still widespread in Scotland. For instance, in two clinical disease scenarios (mastitis and metritis), approximately 30% of the farmers reported feeding milk to calves following antimicrobial administration to those cows. This practice seems to be reduced compared to past years in the UK [14, 39] or compared to other countries [40], but it still represents a potential risk for AMR emergence. Indeed, studies exploring the impact of feeding milk containing antimicrobial residues to calves demonstrated the selection of antimicrobial-resistant bacteria [41, 42].

As a result of the initiatives and awareness-raising actions proposed by the government and international organisations, antimicrobial sales in livestock have decreased over the last decade [43]. The most recent version of the UK Veterinary Antibiotic Resistance and Sales Surveillance (UK-VARSS, 2021) reported a reduction of 68% in cattle injectable HP-CIAs between 2016 and 2020 [43]. Almost all participants of this study (90%) reported having reduced their AMU in previous years, indicating a more promising result than Jones et al., where only 37% of respondents indicated a less frequent AMU compared to the previous year [13]. Nevertheless, many farmers believed that reducing AMU was complicated, with limited facilities (e.g., lack of isolation pen for sick animals, inadequate space allowance leading to high stocking density) and knowledge of appropriate AMU being the main barriers. Also, they indicated time and budgetary constraints as important limitations to decreasing AMU. For instance, being unable to guarantee proper hygiene practices due to time and workforce shortage (e.g., cleaning, bedding, regular mastitis detection) and poor financial means to reinvest in disease prevention (e.g., housing, ventilation). Other studies reported that economic constraints, tight profit margins, and inadequate facilities hinder farmers from improving herd health and reducing AMU [12, 19, 22]. Since producers may be discouraged by financial limitations, advisors must demonstrate the effectiveness of simple, low-cost hygiene/management practices to prevent infections. Also, economic rewards and incentives for low antimicrobial users may motivate farmers to implement new strategies and optimise AMU [44].

Regarding future intention, 87% reported planning to decrease AMU in the next five years. Once more, this proportion is higher compared to previous UK studies [13, 14], suggesting that recent antimicrobial stewardship campaigns (e.g., RUMA Targets task force) had positive impacts on farmers’ intentions [45].

Understanding farmers’ attitudes toward AMR is essential to implement specific strategies and achieving responsible AMU. In our survey, we found a wide variety of attitudes, with up to 25% of respondents disagreeing with some of the statements regarding AMR. These results suggest that farmers’ awareness of AMR is varied and complex, and veterinarians and advisors should consider this before recommending interventions. Tailored strategies would be more beneficial than a generic approach, as each producer’s willingness to reduce AMU is different [46]. For instance, farmers arguing the global health consequences of AMR and with no intention to decrease farm AMU would benefit first further education on the subject and awareness-raising initiatives. In contrast, other farmers may be receptive to more in-depth AMU practice changes. In this study, most respondents (approximately 90%) endorsed the importance of decreasing AMU on UK dairy farms. However, this finding conflicts with the fact that only half of them agreed on the over-reliance on antimicrobials, or expressed concerns about AMR. Then, for some farmers, there may be a mismatch between the intention to reduce AMU and the actual recognition of over-using antimicrobials. As suggested by other studies in the UK [12, 47], this may indicate that dairy producers do not perceive AMR as a current risk for their farm or something they have already experienced. Instead, they may be more concerned about daily challenges, such as welfare, poor housing conditions, and productivity [28]. Respondents generally agreed on the association between AMU and AMR in livestock. However, a smaller proportion acknowledged the potential impact of dairy cattle AMU on global health. Scepticism about the contribution of AMU in cattle to human AMR was reported in other studies [12, 26, 48]. Renunciation of responsibility can be an important barrier to improving AMU on dairy farms. It is difficult to convince farmers to change their practices if they ignore the adverse effects of imprudent AMU. Raising awareness about the potential risk of AMR for global health may be an effective strategy to influence farmers’ AMU behaviour. Indeed, we found that farmers lacking knowledge regarding AMR meaning expressed fewer concerns about AMR.

Many farmers in this study believed that more training is essential for reducing AMU. Self-efficacy, or perceived behavioural control, depends on a person’s belief that they are able to accomplish a task [28, 29]. If farmers believe they possess the knowledge and the skill to achieve reduced AMU, they would be more likely to overcome habitual behaviour and implement new strategies. This study highlights the importance of providing dairy producers with the tools they need to reach responsible AMU, such as regular training on prudent AMU and antimicrobials administration, guidance from veterinarians or external advisors, and assessment of the outcomes (e.g., regular farm AMU monitoring and review of the goals achieved).

Several limitations may have influenced the results of this study. Self-selection bias due to voluntary participation in the online survey was possible, with overrepresentation of farmers with a particular interest in the topic or a higher educational level. Farmers that had experienced issues with lack of efficacy of antimicrobials may have been more motivated to participate. We noted that 33% of respondents had a university degree; however, we were unable to access data to verify whether the educational level of respondents was representative of Scottish dairy farmers. Participants not involved in antimicrobial administration may not have been fully aware of the practices and AMU on the farm. All farmers in this study were working with milking cows, suggesting that these results may not apply to different dairy sectors, such as heifer rearing farms. In the survey, social desirability bias may happen when respondents give socially accepted answers that do not guarantee the truth. In this study, anonymity was guaranteed, so social desirability bias was likely limited. Despite a low response rate, as expected for non-random online surveys, the sample represented a good proportion of the targeted group (Scottish dairy farmers). Regarding the design of the survey, many of the questions (e.g., attitudinal statements, clinical scenarios) were based on focus group/workshop findings and on authors’ experience, which may have introduced bias.

This survey represents the first time that practices and knowledge around AMU and AMR have been investigated in the Scottish dairy sector. The results show that significant progress has been achieved regarding AMU and best practices implementation (e.g., SDCT, discarding waste milk) when comparing the results to previous UK surveys [14, 39]. Yet, awareness and attitudes towards AMR varied among farmers. To help veterinarians and advisors tackle AMR in Scottish dairy herds, future research should focus on identifying the factors influencing farmers’ intentions to reduce AMU.

## Electronic supplementary material

Below is the link to the electronic supplementary material.

Supplementary Material 1

Supplementary Material 2

## Data Availability

The datasets used and/or analysed during the current study are available from the corresponding author on reasonable request.
