# Supplementary material for: Antimicrobial usage and resistance in scottish dairy herds: a survey of farmers’ knowledge, behaviours and attitudes
Source: BMC Vet Res. 2023 May 19;19:72. doi: 10.1186/s12917-023-03625-0 (PMC10197045; doi:10.1186/s12917-023-03625-0)
Supplement: Supplementary file 1 — Supplementary Material 1 [file 12917_2023_3625_MOESM1_ESM.docx]

**Focus group questions – open discussion:**

1. How do you feel about antimicrobial resistance?
2. Do you think there is too much reliance on antimicrobial usage on dairy farms?
3. Do you think some antimicrobials work less effectively than in the past?
4. Which advantages and disadvantages are associated with reducing antimicrobial usage?
5. Do you think your farm antimicrobial usage has changed in recent years?
6. Do you expect your antimicrobial usage to change over the next 5 years?

**Workshop questions pools and answer choices:**

1. How concerned are you about antimicrobial resistance on dairy farms?
   - Very concerned
   - Concerned to some extent
   - I don’t think about it much either way
   - Not concerned at all
2. Which is your main source of information regarding correct antimicrobial usage?
   - Veterinarians
   - Websites
   - Farming articles
   - Guidance from milk buyer
   - Other farmers
   - Not sure/other
3. Do you think antimicrobial usage has changed on dairy farms over the last few years?
   - It has increased
   - It is about the same
   - It has decreased
   - Not sure
4. Which of the following diseases is the main reason for antimicrobial usage on your farm?
   - Mastitis
   - Calf pneumonia/diarrhea
   - Lameness
   - Metritis/post-calving disease
   - Dry cow therapy
   - Other
5. Which is your most frequently used antimicrobial?
   - Beta-lactams/Streptomycin (Pen-Strep®)
   - Oxytetracycline (Alamycin®)
   - Tylosin (Tylan®)
   - Ceftiofur (Naxcel®)
   - Other
6. Do you think it is possible to reduce antimicrobial use on dairy farms?
   - Yes, easily
   - Yes, possible but with some barriers
   - No, too difficult
   - Not sure
7. Which one of the following would be the main barrier in reducing antimicrobial use on your dairy farm?
   - Limited knowledge/training
   - Limited time/labour
   - Limited finances
   - Lack of staff compliance
   - Not sure/other
8. Which of the following would be the main advantage in reducing antimicrobial use on dairy farms?
   - Reduced antimicrobial cost
   - Reduced antimicrobial resistance
   - Increased consumer confidence
   - Increased milk safety
   - Not sure
9. Which of the following would be the main disadvantage in reducing antimicrobial use on dairy farms?
   - Reduced animal welfare
   - Reduced milk production
   - Increased disease/mortality
   - Time consuming/ increased labour
   - Not sure
