# Supplementary material for: Antimicrobial usage and resistance in scottish dairy herds: a survey of farmers’ knowledge, behaviours and attitudes
Source: BMC Vet Res. 2023 May 19;19:72. doi: 10.1186/s12917-023-03625-0 (PMC10197045; doi:10.1186/s12917-023-03625-0)
Supplement: Supplementary file 2 — Supplementary Material 2 [file 12917_2023_3625_MOESM2_ESM.docx]

1. I confirm that I have read and understood the Participant Information Sheet and I consent to the data to be collected and used as described
2. What does “Antibiotic resistance” mean to you in your own words? (Open question)
3. In your opinion, are antibiotics effective against the following pathogenic organisms: viruses, bacteria, and parasites? Tick one option for each pathogen
   - Not effective at all
   - Somewhat effective
   - Very effective
   - Don’t know
4. What effects do antibiotics have? (Tick all that apply)

- Analgesic (reduce pain)
- Anti-inflammatory (reduce inflammation)
- Antipyretic (reduce animal’s temperature)
- Anti-bacterial (kill or inhibit bacteria causing the infection)
- Other

1. Have you ever heard of the RUMA (Responsible Use of Medicine in Agriculture) guidelines for the responsible usage of antibiotics in cattle production?

- Yes
- No

**5.a**. If yes, how familiar are you with the guidelines?

- Not familiar at all
- Somewhat familiar
- Very familiar

1. Over the last month, how many times did you see your vet for a routine visit (e.g. fertility, foot trimming/mobility scoring, disbudding, calves check etc...)

- Never
- Once
- More than once
- Once every week or more

1. Over the last month, how many times did you see your vet for an emergency or non-routine visit (e.g. calving, milk drop, pneumonia, lameness, mastitis, sick calf etc...)?

- Never
- Once
- More than once
- Once every week or more

1. How often do you consult your vet before using an antibiotic?

- Never
- Sometimes
- Most of the time
- Always

1. Have you ever spoken with your vet about antibiotic resistance?

- Yes
- No

**9.a**. If yes, how often do you speak about it approximately?

- Annually
- Every six months
- Monthly
- At every visit

1. Have you ever sought advice on antibiotic usage from any of the following sources and how confident are you in the information you received: farming articles, other farmers, web, milk buyer, veterinarian? Tick if the following sources are used and the level of confidence in them

- Not used
- Used with low confidence
- Used with medium confidence
- Used with high confidence

1. How important to you is the opinion of the following people around antibiotic reduction: veterinarian, other farmers, milk buyer, consumers, colleagues, family? Tick the degree of importance for each person/people

- Not important
- Somewhat important
- Very important

1. Which is your most frequently used antibiotic?

- Penicillin/Amoxycillin
- Oxytetracycline
- Tylosin
- Ceftiofur
- Other

1. Which of the following diseases is the main reason for antibiotic usage on your farm? Rank them from 1 to 6, with 1 being the most common reason for usage and 6 being the least

- Mastitis
- Calf pneumonia
- Calf scour
- Lameness
- Post-partum diseases
- Dry cow therapy

1. Do you have any practices in place on your farm to reduce the usage of antibiotics?

- Yes. Which practices do you use? (Open question)
- No

1. Do you have written protocols regarding the choice of antibiotics on farm?

- Yes
- No, but I am considering developing it in the future
- No, and I do not intend to do it

1. What is the most important reason for calling the vet when you have a sick animal?

- Economic value of the animal
- Previous treatment unsuccessful
- Several animals involved
- Animal welfare
- Others

1. What is the main reason for not calling the vet when you have a sick animal?

- Cost
- Delay in treating animals
- Vet visit means additional work
- I have enough experience
- Others

1. Which factors do you consider important when choosing an antibiotic: cost, vet advice, previous experience, withdrawal period? Tick which factors you consider and the related level of importance for you

- Not considered
- Considered with low importance
- Considered with medium importance
- Considered with high importance

1. How frequently do you send samples for culture and sensitivity (milk, faeces, nasal swabs) before using antibiotics?

- Never
- Occasionally
- Regularly

19.a. If answered never or occasionally, why?

- Too expensive
- It takes time before having the results
- I am not sure about the benefit
- Inconclusive results occur too often
- Other

1. Do you use selective dry cow therapy on your farm?

- Yes. In which approximate percentage of milking cows do you use antibiotics?
- No, but I am considering doing it in the future
- No, and I do not intend to do it

1. How has antibiotic usage on your farm changed over the last few years?

- Less
- Same
- More

**21.a.** If your antibiotic usage has decreased, was it difficult?

- Yes. Which were the main barriers? (Open question)
- No
- I do not know

**12.b.** If your antibiotic usage did not change, do you think it would be difficult to reduce it?

- Yes. What are the main barriers? (Open question)
- No
- I do not know

1. How do you expect your antibiotic usage to change over the next five years?

- Less
- Same
- More

1. How much would the following factors influence your decision to reduce antibiotic usage on farm: reduced antibiotics cost, reduced animal antibiotic resistance, reduced human antibiotic resistance, minimise the risk of antibiotic residues in milk, meet milk buyer standards, more consumer confidence? Tick the degree of influence for each factor

- No influence
- Some influence
- A lot of influence

1. How much do the following factors concern you about reducing antibiotic usage on farm: increased animal disease/death, decreased profitability, decreased milk production, reduced animal welfare, increased costs (e.g., new facilities required)? Tick the degree of concern for each factor

- Not concerning
- Somewhat concerning
- Very concerning

1. Scenario 1: Milking cow: sign of mild mastitis (milk modified, udder inflamed, no fever, no systemic signs)

**25.a.** What would you do first?

- Call the vet
- Administer an intramammary antibiotic tube. Which one? (Open question)
- Administer a systemic antibiotic. Which one? (Open question)
- Take a milk sample for culture
- Monitor the cow
- NSAIDS/fluids
- Other

25.b. If you treat the cows with antibiotics, where would you record the treatment? (Tick all that apply)

- I do not record
- Treatment book
- Mark the cow
- Computer
- Other

25.c. If you treat the cows with antibiotic, what do you do with the milk?

- Throw it away
- Feed to all calves
- Feed to some calves but not replacement heifers
- Other

1. Scenario 2: 1-week-old calf: diarrhoea, no fever, slightly dehydrated, normal appetite

26.a. What would you do first?

- Administer an antibiotic. Which one? (Open question)
- NSAIDS/fluids
- Call the vet
- Other

26.b. If you would not administer an antibiotic, what is the reason? (Tick all that apply)

- It’s not worth treating calves
- The disease is not severe enough
- Just one calf is affected
- I want to use antibiotics responsibly
- I don’t usually use antibiotics for calf scour
- It is not advised in my written protocols

26.c. What would you do to limit the spread to other calves? (Tick all that apply)

- Nothing
- Isolate the sick calf
- I use specific tools/equipment for the sick animal
- Feed the calf last
- Do a prophylactic treatment to other calves
- Other

1. Scenario 3: Cow: 10 days post-partum, smelly uterine discharge, temperature 39.5°C

27.a. What would you do first?

- Administer an antibiotic. Which one? (Open question)
- NSAIDS/fluids
- Call the vet
- Monitor the cow
- Other

27.b. If you treat the cows with an antibiotic, why would you choose it? (Tick all that apply)

- I Follow my written treatment protocol
- I do what the vet previously advised to me
- Because it is cheap
- Because I am familiar with this drug
- Other

27.c. If you treat the cows with antibiotic, would you use the milk to feed the calves?

- Yes
- No
- Only to some calves

1. Scenario 4: Six calves aged 1–2 months: cough, nasal discharge, fever (temperature > 39.5°C)

28.a. What would you do first?

- Administer an antibiotic. Which one? (Open question)
- NSAIDS
- Call the vet
- Monitor
- Other

28.b.If you treat the calves with an antibiotic, how do you know which calves have been treated? (Tick all that apply)

- I do not record
- Treatment book
- Mark the calves
- Computer
- Other

28.c. If you treat the calves with an antibiotic, how long do you treat the calves for?

- I follow my written protocols
- I follow previous vet advice
- I follow the instructions on the drug leaflet
- Until the calves look well
- Other

1. Diarrhoea in 20% of young calves (1–3 weeks old) over the last month, and a few of them died

29.a. What do you think is the most efficient action to take in order to prevent the other animals from getting infected?

- Vaccinate cows
- Take a faecal sample to identify the infectious agent
- Do a prophylactic treatment
- Other

29.b. Which other action would you take to reduce the spread of the infection on farm? (Tick all that apply)

- Nothing
- Cleaning and disinfecting the pens
- Ensure colostrum intake/quality
- Feeding sick animals at the end
- Isolate sick animals
- Other

1. Milking cow: sudden lameness in one hind limb

30.a. What would you do first?

- Administer an antibiotic. Which one? (Open question)
- Call the vet
- NSAIDS
- Wait for the foot trimmer
- Examine the foot

30.b. If you choose to treat the cow with an antibiotic, how long would you treat the animal for?

- Until improvement of the lameness
- What worked in my experience
- What is recommended in my protocols
- Other

1. Milking cow: sudden milk drop and fever (temperature = 39.9 °C)

31.a. What do you do first?

- I administer an antibiotic. Which one? (Open question)
- I take a milk sample
- I call the vet
- NSAIDS/fluids
- Monitor
- Other

1. It is important to reduce antibiotic usage on UK dairy farms

- Strongly agree
- Agree
- Neither agree nor disagree
- Disagree
- Strongly disagree

1. Nowadays, there is too much reliance on antibiotic usage on dairy farms in the UK

- Strongly agree
- Agree
- Neither agree nor disagree
- Disagree
- Strongly disagree

1. Decreasing antibiotic usage in dairy farms could help reducing antibiotic resistance in livestock

- Strongly agree
- Agree
- Neither agree nor disagree
- Disagree
- Strongly disagree

1. Decreasing antibiotic usage in dairy farms could help reducing antibiotic resistance in humans

- Strongly agree
- Agree
- Neither agree nor disagree
- Disagree
- Strongly disagree

1. Some antibiotics work less effectively than in the past

- Strongly agree
- Agree
- Neither agree nor disagree
- Disagree
- Strongly disagree

1. Farmers require more training on antibiotic usage

- Strongly agree
- Agree
- Neither agree nor disagree
- Disagree
- Strongly disagree

1. Farm biosecurity and vaccination can reduce antibiotic usage

- Strongly agree
- Agree
- Neither agree nor disagree
- Disagree
- Strongly disagree

1. It is important to have protocols for antibiotic usage on farm

- Strongly agree
- Agree
- Neither agree nor disagree
- Disagree
- Strongly disagree

1. It is important to keep treatment records on farm and review antibiotic usage regularly

- Strongly agree
- Agree
- Neither agree nor disagree
- Disagree
- Strongly disagree

1. It is important to always respect the prescribed duration course of antibiotic

- Strongly agree
- Agree
- Neither agree nor disagree
- Disagree
- Strongly disagree

1. It is important to have hospital pens to isolate sick animals and avoid the spread of the diseases

- Strongly agree
- Agree
- Neither agree nor disagree
- Disagree
- Strongly disagree

1. It is important to always respect the withdrawal period of treated animals before slaughter or including the milk in the bulk milk tank

- Strongly agree
- Agree
- Neither agree nor disagree
- Disagree
- Strongly disagree

1. I am worried about antibiotic resistance on UK dairy farm

- Strongly agree
- Agree
- Neither agree nor disagree
- Disagree
- Strongly disagree

1. What is your age?

- 18-35
- 36-50
- More than 51
- Prefer not to say

1. What is your sex?

- Male
- Female
- Prefer not to say

1. How many years of experience (post-school age) in dairy farming do you have?

- Less than 5
- 6-20
- 21-40
- More than 41

1. What is your highest level of education?

- High school
- Agricultural college
- University
- Other

1. Is your dairy farm:

- Conventional
- Organic

1. Please provide an approximate number of dairy animals on your farm:

- Milking/dry cows
- Replacement heifers (weaned)
- Calves unweaned (male and female)
- Dairy bulls
- Other

1. Do you have any disease-free control accreditation? (Thick all that apply)

- No
- BVD
- Johnes
- Lepto
- IBR
- Other

1. Have you bought new animals on to the farm over the last year?

- Yes
- No

1. Do you have other species/livestock types on farm?

- No
- Sheep
- Beef
- Other

1. What is your role in the enterprise?

- Owner
- Dairy manager
- Other

1. Please give an approximate value for each of the following questions.

- What is the average milk production par cow (litres)?
- What is the total milk production on the farm par year (litres)?
- What is the geometric average somatic cell count (cells/ml)?

1. Who is your milk buyer? (Optional question)
